# Source Tracking *Mycobacterium ulcerans* Infections in the Ashanti Region, Ghana

**DOI:** 10.1371/journal.pntd.0003437

**Published:** 2015-01-22

**Authors:** Charles A. Narh, Lydia Mosi, Charles Quaye, Christelle Dassi, Daniele O. Konan, Samuel C. K. Tay, Dziedzom K. de Souza, Daniel A. Boakye, Bassirou Bonfoh

**Affiliations:** 1 Parasitology Department, Noguchi Memorial Institute for Medical Research, University of Ghana, Legon, Ghana; 2 Centre Suisse de Recherches Scientifiques en Côte d’Ivoire, Adiopodume, Côte d’Ivoire; 3 Clinical Microbiology Department, School of Medical Sciences, Kwame Nkrumah University of Science and Technology, Kumasi, Ghana; 4 Biochemistry, Cell and Molecular Biology Department, University of Ghana, Legon, Ghana; Swiss Tropical and Public Health Institute, SWITZERLAND

## Abstract

Although several studies have associated *Mycobacterium ulcerans* (MU) infection, Buruli ulcer (BU), with slow moving water bodies, there is still no definite mode of transmission. Ecological and transmission studies suggest Variable Number Tandem Repeat (VNTR) typing as a useful tool to differentiate MU strains from other Mycolactone Producing Mycobacteria (MPM). Deciphering the genetic relatedness of clinical and environmental isolates is seminal to determining reservoirs, vectors and transmission routes. In this study, we attempted to source-track MU infections to specific water bodies by matching VNTR profiles of MU in human samples to those in the environment. Environmental samples were collected from 10 water bodies in four BU endemic communities in the Ashanti region, Ghana. Four VNTR loci in MU Agy99 genome, were used to genotype environmental MU ecovars, and those from 14 confirmed BU patients within the same study area. Length polymorphism was confirmed with sequencing. MU was present in the 3 different types of water bodies, but significantly higher in biofilm samples. Four MU genotypes, designated W, X, Y and Z, were typed in both human and environmental samples. Other reported genotypes were only found in water bodies. Animal trapping identified 1 mouse with lesion characteristic of BU, which was confirmed as MU infection. Our findings suggest that patients may have been infected from community associated water bodies. Further, we present evidence that small mammals within endemic communities could be susceptible to MU infections. *M. ulcerans* transmission could involve several routes where humans have contact with risk environments, which may be further compounded by water bodies acting as vehicles for disseminating strains.

## Author Summary

Buruli ulcer is a skin disease, which is endemic in over thirty countries, mostly in West Africa, with affected populations being largely rural. The causative organism, *Mycobacterium ulcerans* (MU), is an environmental mycobacterium and although transmission is unclear, frequent exposure to these MU-contaminated environments have been suggested as risk factors. We conducted this study on the premise that if patients are infected from MU-contaminated water bodies, then the genotype of MU strains in these patients should be identical to those in their community associated water bodies and wetlands. Using Variable Number Tandem Repeat (VNTR) as a genetic tool, we determined the genotypes of MU from both water bodies and patient samples. Comparison and overlap of these genotypes, within each community, suggest that patients were possibly infected from at least one water body. Additionally, we present evidence that small mammals within endemic communities could be susceptible to MU infections and may be acting as reservoirs. Our findings suggest that future ecological and molecular studies in the hope of elucidating a definite transmission route, should focus on source-tracking MU infections to community associated risk environments while employing a OneHealth approach in the process.

## Introduction

Buruli ulcer (BU) is a necrotizing skin disease which has been reported in over thirty countries. The most endemic countries include Ghana, Togo, Cote d’Ivoire and Benin with affected populations significantly being rural [1]. *Mycobacterium ulcerans* (MU), the causative agent of BU is an environmental mycobacteria. The mode of transmission to humans is still not clear although a few hypotheses have been advanced and tested [2–4]. Relying on advances in environmental microbiology and genotyping tools to identify habitats and reservoirs of *M. ulcerans* persistence and proliferation could aid in such transmission studies [5, 6]. Human-to-human transmission is rare [7] and infection seems to be high among people with frequent contact to slow moving water bodies or wet lands, in endemic communities [5, 8].Within an aquatic environment, *M. ulcerans* can be found at the air-water interface, form biofilm on surfaces and probably occupy microhabitats not directly exposed to light but aerated [9]. From these biotopes, it is possible for the bacteria to infect susceptible hosts [10]. The viability of MU from environmental sources was proven with successful cultivation of the bacterium from an aquatic insect and subsequent establishment of infection in a mouse model [11]. Additionally, molecular data have correlated abundance of MU DNA from these environments with increasing BU cases [5]. We posit that patients are infected from MU-contaminated water bodies, hence MU genotypes from both sources should be identical. Earlier efforts have focused on comparing and differentiating human isolates within and from different geographical origins leaving out the environmental component [12–14].

An emerging development on the transmission of MU is the role small mammals could be playing in the ecology of the pathogen. *M. ulcerans* infection with clinical presentations similar to those in humans have been observed in koalas [15], possums [16, 17] and in armadillos [18], in studies conducted in Australia. A study conducted by Durnez *et al*. in Benin, where small mammals were trapped and analyzed for mycobacterial infections, detected several species of mycobacteria but not *M. ulcerans* [19]. Experimental studies in Ghana have also shown indigenous grasscutters, *Thryonomys swinderianus*, to be susceptible to *M. ulcerans* infection [20]. Small mammals, living in close proximity to humans and commonly hunted animals, like grasscutters, rabbits and rats could therefore be potential reservoirs of *M. ulcerans*.

Application of VNTR typing has revealed genetic differences among MU isolates collected from different patients and geographical regions [6, 14, 21–25]. Hilty *et al*. [14], identified three pathogenic MU genotypes in Ghana using this tool. Their findings were corroborated by ecological studies, investigating the distribution of *M. ulcerans* in endemic and non-endemic communities in Ghana [6]. The addition of other polymorphic loci will therefore increase the discrimination power in differentiating intra-species variation.

Transmission of environmental mycobacteria is dependent on the overlapping habitats of the pathogen and humans [26]. Major overlap occurs in water where humans are exposed to mycobacteria through drinking, swimming, and bathing [26]. Thus, we source-tracked human MU infections to 10 water bodies, in four BU endemic communities, in Ghana. Using VNTR as an identifying tool, we uncovered additional genotypes and showed that patients were most likely infected from the water bodies they were frequently exposed to. We also assessed the role of small mammals as reservoirs of MU and suggest that they may be susceptible to MU infections and/or act as reservoirs. Using the ‘OneHealth’ concept, which seeks to define, manage and prevent diseases using a holistic approach of human, animal and environmental importance, we discussed a plausible transmission model, suggesting possible routes of MU infections from the environment.

## Materials and Methods

### Ethics statement

Ethical approval for patient recruitment into the study was sought and approved from the institutional review board of the Noguchi Memorial Institute for Medical Research (NMIMR), University of Ghana, (FWA 00001824; IRB00001276; IORG0000908). This also covered the administration of questionnaires. All participants signed a written informed consent form before recruitment into the study. All suspected cases were confirmed by PCR and results sent back to the District Health Directorate for treatment to commence. Approval for animal trapping and collection was under the permit of the Ashanti Regional Ministry of Health and the Wildlife Division of the Forestry Commission of Ghana (FCWD/GH-02), following its guidelines on animal husbandry. Dissection and harvesting of animal organs was adapted from Durnez *et al*. [19].

### Selected communities and water bodies

The study was carried out in Numereso, sub-district within the Amansie Central District of the Ashanti region, Ghana. The four selected communities for this study were Wromanso (**N**06.03256 **W**001.89761), Bepotenten (**N**06.09213 **W**001.96604), Monia-Gyaman (**N**06.05113 **W**001.92242) and Sukuumu (**N**06.05190 **W**001.94341). These communities were selected based on reported BU prevalence of 12.1, 7.8, 6.6 and 6.4 (per 1000 persons) for Bepotenten, Sukuumu, Monia-Gyaman and Wromanso respectively (Amansie District Health Directorate records). There was at least one working borehole with pump in each community, but inhabitants still used water from water bodies, for domestic and agricultural activities (S1 Table). We sought to identify among other things, livelihood strategies, hunting and animal rearing in households and perception on the modes of BU transmission, using a questionnaire we developed.

### Environmental sampling

Environmental sampling followed procedures described by Williamson *et al*. [6] with slight modifications. Soil, water filtrands and detritus were collected in triplicates and biofilm in quintuplicates. A sterile scalpel was used to collect about 5g of soil from the water floor and two from the riparian zone 5m apart into a 15ml falcon tube (BD Biosciences). This was preserved in 96% ethanol (Pharmacos). Detritus consisted of dead leaves, stems and grass blades within the water body. These were cut into a 15ml falcon tube and also preserved in 96% ethanol. Biofilm were taken from the surfaces of stems and leaves of dominant aquatic vegetation. Briefly, these parts were cut into Ziplock bags,100ml of double distilled water was added, sealed and the biofilm material was dislodged by rubbing the bag vigorously several times. About 50ml of the resulting suspension was then poured out into a falcon tube for later analysis. For water filtrand, about 2L of water was scooped from the surface of the water body. Fifty milliliters of this was filtered through a 0.45µm nitrocellulose filter (Whatman Inc). The water was then pumped through until the built resistance could not be overcome. The nitrocellulose filter was then removed and wrapped completely in aluminum foil. All samples were kept cool and transported to the laboratory where they were preserved at 4°C until processing. All materials were washed and decontaminated with 70% bleach and, sterilized with 90% ethanol and DNA away (Molecular BioProducts) between sampling sites.

### Sample collection from suspected BU patients

Active case searches were organized in the four communities and clinical samples taken from all suspected BU cases for confirmation and for the study. All participants signed a consent form before recruitment into the study. All suspected cases were confirmed by PCR and results sent back to the District Health Directorate for treatment to commence. Samples taken which were stored in a cooler included fine needle aspirates (FNA) for nodules and swabs for open lesions.

### Small mammal trapping and dissection

Sherman livetraps (H.B. Sherman Traps, Inc.) were used in all small mammal collections. Traps, baited with a mixture of dried fish, groundnut paste and flour were set randomly in selected houses, farms and near water bodies. A total of 100 traps were set per community per night. Successful traps were labeled with the GPS coordinates of the site and all traps were washed with bleach between communities. Trapped animals were euthanized with chloroform and examined for external lesions and swellings before dissection. Organs including heart, lungs, liver, stomach, small intestines, caecum, kidneys, and spleen were harvested into separately labeled vials (2ml screw-cap tubes). Additionally, anal swabs, lesion swabs and tissue biopsies of lesions, if present, were taken. Animal carcasses were disinfected and buried 1.5ft below the ground. This paper presents data on the five animals with external lesions.

### Sample processing for laboratory analyses

Biofilm samples were concentrated with an optimized protocol developed in the lab. Briefly, the 50ml falcon tubes containing the biofilm in suspension were spun at 12,000 rpm for 5 minutes in a High Speed Refrigerated Centrifuge (Suprema 21, TOMY) at 4°C. Twenty milliliters of the supernatant was carefully decanted off. This procedure was repeated at 13,000 and 14,000 rpm and each time discarding 5mL of the resulting supernatant. The remaining 10ml was preserved at 4°C until further use. All other environmental samples were processed as previously described [7]. FNAs and swabs from patient samples were processed for culture, microscopy, DNA extraction and PCR using protocols from other studies [6, 27, 28]. The samples were cultured in two ways. In one set, serial dilutions were performed, plated on LJ media slants and incubated at 32ºC. In another set, samples were decontaminated using the Modified Petroffs method and plated on LJ media slants. Processing of animal samples was carried out in a biosafety cabinet (Clean Bench, Hitachi). About a half of each organ sample (entire biopsy was used) was homogenized on a glass slide using a scalpel. The homogenized tissue was then scraped into a vial containing 1ml of 1X PBS. The homogenate was then vortexed vigorously and 250µl was used for extraction.

### DNA extraction, PCR and sequence analyses

DNA extraction for human and animal samples were performed using the Qiagen Dneasy blood and tissue kit (QIAGEN) following the manufacturer’s protocol. For environmental samples, DNA extraction followed the protocol described by Williamson *et al*. [6].

Negative and positive controls were included for each PCR run. Additionally, for environmental samples spiked samples (2.5µl each of, positive control and an environmental extract) were included to check for inhibitions. Bovine Serum Albumin (Promega) was also added to environmental samples to relieve PCR inhibition in the amplification of all target loci. All PCR reactions were performed in a 2720 Thermal Cycler (Applied Biosystems). All primer sequences used in this study are listed in S2 Table. To identify environmental mycobacteria, samples were first screened using mycobacterial 16S *rRNA* primer as previously described [29]. Samples were then screened for the insertion sequence *IS2404* PCR in a nested PCR, adapted from Ablordey *et al*. [30]. New primer sets were designed to amplify a 476bp product on the *Mls*A domain, encodes enoyl reductase (ER), of pMUM001 plasmid. This was performed in a 25µl reaction containing 1X PCR buffer (Promega), 1.5mM MgCl_2_, 400µM each of deoxyribonucleotide (Promega), 160nM each of forward and reverse primers, 1U GoTaq polymerase (Promega) and 5µl of genomic DNA. Reaction was cycled at 95°C for 3mins followed by 40 cycles each of, denaturation at 94°C for 30s, annealing at 63°C for 35s and extension at 72°C for 45s. Final extension was at 72°C for 10mins and reaction held at 4°C.

PCR for VNTRs was adapted from Williamson *et al*. and Hilty *et al*. [5, 14] with slight modifications. Allelic profiles used were in the order: MIRU1, Locus 6, ST1, Locus 19. Briefly, thermal cycling for all four VNTR loci were increased by additional 5 cycles. Seven microliters (7uL) of PCR products were run on a 2% agarose gel (Sigma-Aldrich), stained with ethidium bromide (Sigma-Aldrich) and band sizes were estimated with 100bp ladder. Repeat numbers for all VNTR loci were calculated based on published data [6, 14, 21, 23–25].

Representative amplicons of *IS2404*, ER, 16S and VNTRs were confirmed with sequencing. VNTR-profiling was performed for only *IS2404* positive samples and repeats confirmed with sequencing randomized samples. PCR products (40uL), with varying repeat sizes for each locus, were sent for sequencing (Macrogen Inc, Netherlands). Multi sequence alignments (MSA) and phylogenetic analyses were performed within MEGA V5 [31]. Representative sequences for loci including *IS2404*, ER, MIRU1, ST1, Locus 6 and Locus 19 have been deposited in GenBank under the following accession numbers, KM459595, KM459596, KM459597, KM459598, KM459599, KM459600, KM459601, KM459602, KM459603 and KM459604.

## Results

### Profile of selected communities

**Figure 1:**
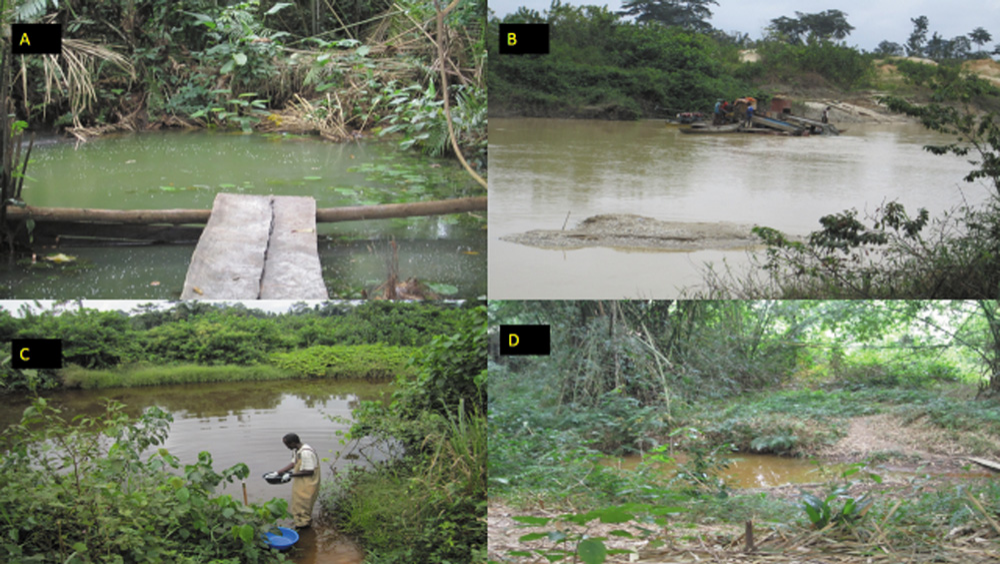
Four water bodies that were sampled. A) Twingun 2 pond at Sukuumu; B) illegal mining activities (galamsey) on the Offin River at Monia-Gyaman; C) Nkotia stream at Sukuumu; D) Bebonu pond at Wromanso.

Majority of the inhabitants in all four communities were farmers (S3 Table) but some youth were into small scale surface gold mining (galamsey) mainly along the Offin River (Fig. 1), which runs through all four communities. Preliminary analysis showed that although about 50% of inhabitants in a community used nearby surface water bodies for various purposes, swimming and bathing in these water bodies were the only activities (S1 Table) that were associated with an increased risk for BU infection (unpublished data). This informed our choice of specific water bodies to sample. In total, ten water bodies were selected after active case surveillance, where 2, 4, 1 and 7 cases were detected in Wromanso, Monia-Gyaman, Bepotenten and Sukuumu respectively.

### Case confirmation

Both microscopy and culture were attempted for FNA and swabs. However, acid-fast bacilli were not detected. There was over growth of other bacteria in the set that was serially diluted but not decontaminated. No growth was observed in the decontaminated set. Case confirmation was therefore based only on PCR (Table 1).

**Table 1:** MU confirmation and VNTR profile of MU detected in human samples.

**Amansie Central**		**Tests**							
		**Diagnosis**			**VNTR allelic profiles**				**Genotype**
Communities	Test ID	16S	IS2404	ER	MIRU1	L 6	ST1	L 19	
Wromanso	FW1	Pos	Pos	Pos	1	2	2	2	Z
	FW2	Pos	Pos	Pos	1	2	2	1	Y
Monia-Gyaman	SM1	Pos	Pos	Pos	1	1	2	2	X
	SM2	Pos	Pos	Pos	1	1	2	1	W
	FM3	Pos	Pos	Pos	1	2	2	1	Y
	FM4	Pos	Pos	Pos	1	1	2	2	X
Bepotenten	FB1	Pos	Pos	Pos	1	2	2	1	Y
	FB2	Neg	Neg	Neg					
Sukuumu	FS1	Pos	Pos	Pos	1	1	2	2	X
	FS2	Pos	Pos	Pos	1	2	2	1	Y
	FS3	Pos	Pos	Pos	1	1	2	0	UA
	FS4	Pos	Pos	Pos	1	2	2	2	Z
	FS5	Pos	Pos	Pos	1	1	2	2	X
					1	1	2	1	W
	FS6	Pos	Pos	Pos	1	1	2	0	UA
	FY1	Pos	Pos	Pos	1	2	2	2	Z

UA, unassigned. PCR amplification of Locus 19 was unsuccessful for FS3 and FS6. Locus 6 and Locus 19 were the main determinants for a genotype. Patient, FB2 was negative for MU. Pos, positive, Neg = negative, L6 = locus 6, L19 = locus 19.

### Preliminary detection of MPMs in environmental samples

All environmental samples (N = 140) were first screened for *Mycobacterium spp* using the mycobacterial 16S *rRNA* primers. Positive samples were then tested for *IS2404*. Total sample positivity was 38/140 (27%) and 25/38 (66%) for 16S and *IS2404* respectively (Table 2). The Offin River at Sukuumu had the highest positivity for *Mycobacterium spp* and MPMs. Of the 14 samples from this river, eight contained mycobacteria out of which 5 were confirmed as containing MPMs. Mycobacteria was not detected in all 14 samples from Mon-Offin. At least one sample from all type of water body; **River** (Suk-Offin, Wro-Offin and Bep-Oda), **Stream** (Mon-Nkotia, Mon-Ampoma and Suk-Nkotia) or **Pond** (Wro-Bebonu, Suk-Twingun1 and Suk-Twingun2) tested positive for *IS2404* (Table 2). The Offin River flows close to all four communities, but could not be sampled at Bepotenten as it was inaccessible due to galamsey (illegal surface gold mining) activities at the time of sampling (Fig. 1). Highest positivity for 16S was observed with biofilm 22/50(44%) with the lowest being detritus 2/30 (7%). Differences in matrix positivity was statistically significant (P = 0.0007) when tested with the Chi-square contingency table for independence (Table 2). AFB microscopy showed clumps of bacilli in biofilm samples as compared to single bacilli in detritus (S1 Fig.). Twenty-five of the thirty-eight (66%) 16S positive samples were positive for *IS2404*. All four 16S positive soil samples were *IS2404* positive and 64% of 16S positive biofilm samples tested positive for *IS2404*.

**Table 2:** 16S and IS2404 positivity in sampled water bodies and matrices.

	***N^o^16S positive/total sampled (%)***	***N^o^IS2404 positive/total 16S positive (%)***
***Water bodies***		
*Wro-Offin*	2/14(14%)	2/2(100%)
*Wro-Bebonu*	1/14(7%)	1/1(100%)
*Mon-Offin*	0/14(0%)	0/0(0%)
*Mon-Nkotia*	5/14(36%)	4/5(80%)
*Mon-Ampoma*	3/14(21%)	2/3(67%)
*Bep-Oda*	4/14(29%)	4/4(100%)
*Suk-Offin*	8/14(57%)	5/8(63%)
*Suk-Twingun 1*	3/14(21%)	1/3(34%)
*Suk-Twingun 2*	5/14(36%)	4/5(80%)
*Suk-Nkotia*	7/14(50%)	2/7(29%)
*Total*	38/140 (27%)	25/38(66%)
***Sample matrices***		
*Biofilm*	22/50(44%)	14/22(64%)
*Filter*	10/30(33%)	6/10(60%)
*Detritus*	2/30(7%)	1/2(50%)
*Soil*	4/30(13%)	4/4(100%)
*Total*	38/140(27%)	25/38(66%)

Environmental samples were first screened for mycobacterial 16s rRNA positivity. Positive samples were then screened for IS2404. At each water body, biofilm were collected in quintuplicates. All other samples were collected in triplicates. Biofilm were the most positive, (22/38) for 16S and this difference in matrix positivity was statistically significant (P = 0.0007) using the Chi-square contingency table for independence.

### VNTR analysis of human samples reveals additional *M. ulcerans* ecovars

**Figure 2:**
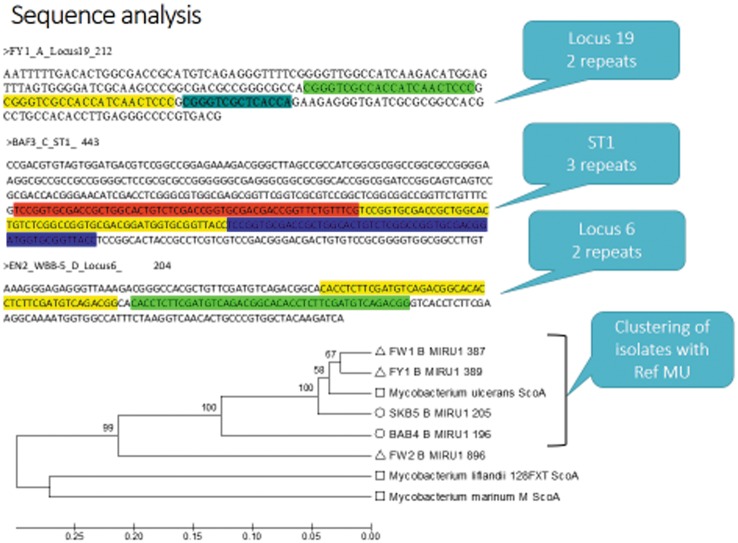
Sequence confirmation of VNTR repeats and phylogeny of MU isolates. The evolutionary history was inferred using the UPGMA method [39]. The optimal tree with the sum of branch length = 1.31639434. The percentage of replicate trees in which the associated taxa clustered together in the bootstrap test (1000 replicates) are shown next to the branches [40]. The reference sequences (represented with square) of the MIRU1 (ScoA gene) orthologs, *M. marinum*, *M. liflandii* and *M. ulcerans* were retrieved from GenBank with accession numbers CP000854.1, CP003899.1 and DQ397533.1 respectively. Sequences of human and environmental samples are represented with triangles and circles respectively. Tandem repeats were analysed using Tandem Repeat Finder [41] and pattern searches in Microsoft Word 2013.

Fifteen suspected patients were included in this study following an active case surveillance. Fourteen were positive for 16S, *IS2404* and ER (Table 1). ER sequence analysis showed >95% similarity to *M. ulcerans* except sample FY1 which had equal identities, 91%, to both *M. ulcerans* and *M. liflandii*. All 14 BU confirmed patients were recommended for treatment. VNTR-PCR was performed for *IS2404* positive samples (14/15) and repeats confirmed with sequencing (Fig. 2). Allelic profiles were written as (MIRU1, Locus 6, ST1, Locus 19). Four genotypes, designated, **W (1, 1, 2, 1)**, **X (1, 1, 2, 2)**, **Y (1, 2, 2, 1)** and **Z (1, 2, 2, 2)**, were observed for human samples. Locus 6 and 19 were the main determinants of a particular genotype because each gave two repeats, 1 or 2. MIRU1 and ST1, invariably gave repeats of 1 and 2, respectively. Four samples showed genotype X, 4 showed genotype Y, 3 typed genotype Z and 2 as genotype W. However, one patient (FS5) had MU infection with two genotypes, X and W (Table 1) with band sizes at 280bp and 340bp at locus 19 (S2 Fig.), differentiating these genotypes. Genotypes for FS3 and FS6 were indeterminate because there was no amplification at locus 19. Interestingly, sample FY1 showed a partial repeat for locus 19 (Fig. 2) leading to the profile Z.

### Environmental samples showed similar *M. ulcerans* genotypes as those confirmed in patients

Six MU genotypes, W, X, Y, Z, A and B, were observed for environmental samples (Table 3), in *IS2404* positive samples. *M. marinum* DL genotype E (1, 2, 1, 2) was observed in a biofilm sample from Twingun pond 2. A biofilm sample, BAB-4, collected from the Oda River, Bepotenten, showed a VNTR profile Y (1, 2, 2, 1) similar to a patient sample, FB1 (Table 1 and S2 Fig.), from the same community. Additional bands were observed, suggesting presence of other MPMs. A similar observation was made for BAF-3, which had ST1 repeat of 3 and was confirmed with sequencing as *M. marinum* M strain (Fig. 2). Also, we observed a unique genotype, OTS (1, 2, 7, ND) in soil and biofilm samples. Genotypes W, X, Y, A, B and E were observed in Sukuumu. Genotypes X and Y, OTS and Y, Z and Y, were observed in Wromanso, Monia-Gyaman and Bepotenten respectively. Twingun 2 pond had the most diverse genotypes (n = 5).

**Table 3:** VNTR analysis of environmental samples.

***Community***	***Water body***	***Type of matrix***	***VNTR allelic profiles***				***Genotypes***
			MIRU1	Locus 6	ST1	Locus 19	
*Wromanso*							
	**Bebonu pond**	**Biofilm**					
		WBB-5	1	2	2	1	Y
		WBB-1	1	2	2	2	Z
*Monia-Gyaman*							
	**Akotia stream**	**Biofilm**					
		MNB	1	2	2	1	Y
		**Detritus**					
		MND	1	2	2	1	Y
		**Soil**					
		MNS	1	2	0	0	UA
	**Ampoma stream**	**Biofilm**					
		MPB	1	2	7	ND	OTS
		**Detritus**					
		MPD	1	2	2	1	Y
*Bepotenten*	**Oda River**	**Biofilm**					
		BAB-4	1	2	2	1	Y
		**Filter**					
		BAF-2	1	2	2	2	Z
		BAF-3	0	2	3	0	UA
*Sukuumu*	**Offin River**	**Biofilm**					
		SOB-5	1	2	2	0	Y-
		SOB-4	1	2	7	ND	OTS
		**Filter**					
		SOF-1	1	2	7	ND	OTS
		**Soil**					
		SOS-3	1	2	2	0	Y-
		SOS-3	1	2	7	ND	OTS
	**Twingun 1 pond**	**Filter**					
		STF	1	ND	1	ND	UA
	**Twingun 2 pond**	**Biofilm**					
		SKB-3	1	1	2	2	X
		SKB-4b	1	1	1	2	A
		SKB-5	1	2	2	1	Y
		SKB-2	1	2	1	2	E
		SKB-4a	3	1	1	2	B
	**Akotia stream**	**Biofilm**					
		SNB-5	1	1	2	1	W
		SNB-2	ND	1	4	1	UA
							

UA, unassigned, OTS, genotypic designation of unidentified MPM, NP, none positive. ND, not done, 0, no amplification. BAF-3 was confirmed as *M. marinum* from BLAST searches. Two water bodies (Wro-Offin and Mon-Offin) are not shown because no VNTR loci was amplified for any of the samples collected. MU presence was independent (P = 0.8081) of the type of water body (river, stream or pond).

### Increased discrimination of VNTR markers suggests heterogeneity of MU strains and corroborates previously reported genotypes in Ghana

Four MU genotypes, W (1, 1, 2, 1), X (1, 1, 2, 2), Y (1, 2, 2, 1) and Z (1, 2, 2, 2) were identified from both human and environmental samples. Comparing them to published MU genotypes, repeat variation (1 or 2), for Locus 6 and 19, were similar (S4 Table). MIRU1and ST1 had conserved repeats of 1 and 2 respectively, for all human MU genotypes in this study. This again was consistent with the published MU genotypes, except for B^b^, C, and the two Amansie MU strains 2 and 3. Amansie MU strains 1, 2 and 3, were profiles from Amansie West, Ghana [14]. This is adjacent to the current study area. Furthermore, Genotype X had similar VNTR profile as MU genotype D [32]. *M. liflandii* with reported genotype F (1, 2, 2, 1) [7] was identical to current study genotype Y (1, 2, 2, 1). Overlapping the VNTR profiles of two Ghanaian isolates, the Amansie West strain 1 and Ghana sequence strain, gives a complete VNTR profile X (1, 1, 2, 2). Other genotypes observed, A (1, 1, 1, 2), B (3, 1, 1, 2) and E (1, 2, 1, 2), were identical to published genotypes, however, they were typed to environmental samples. Phylogenetic analysis clustered human (FW1, FY1 and FW2) and environmental (BAB4 and SKB5) samples with reference *M. ulcerans ScoA* ortholog suggesting genetic relatedness as predicted with the VNTR profiles (Fig. 2). In separate analysis we performed multi sequence alignment of the MIRU repeat consensus sequences from all sequenced amplicons with the corresponding repeat sequence published [14]. We observed a 100% sequence match, thus, supporting the observed clustering of our profiles with reference MU. Similarly, a separate phylogenetic analysis using *IS2404* sequences showed a similar clustering with reported *M. ulcerans* orthologs (S3 Fig.). However, we observed two clusters. Our samples formed one cluster with two *M. ulcerans* orthologs and the other cluster was formed by *IS2404* orthologs of other MPMs.

### Detection and confirmation of MU in animal samples

**Figure 3:**
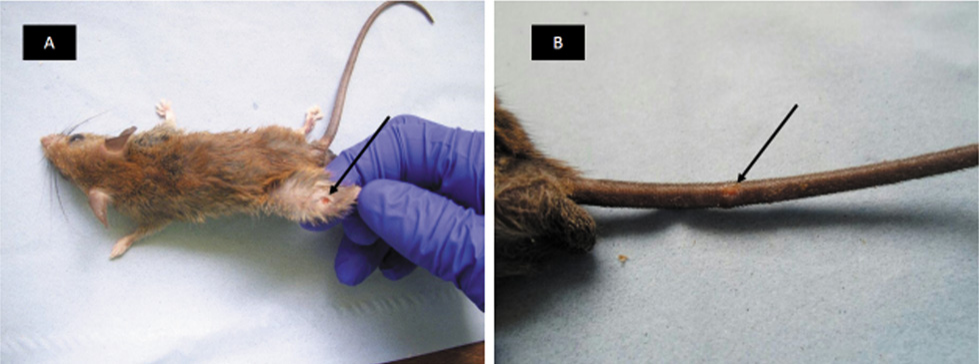
Two mice with lesions characteristic of BU. A) S6, had a lesion <1cm on the left thigh. B) S11, with a lesion <1cm on the tail, Lesion biopsy was positive for 16S *rRNA*, *IS2404* and ER, which was confirmed as MU following sequencing.

A total of 78 small mammals were collected over 4 collection nights. Lesions (Fig. 3) were observed on only five animals (6.41%). Identified lesions were on the thigh, tail (2 animals), ear and abdomen. Table 4 gives the PCR and sequence data analysis for the organs and lesions of the five small mammals. All five had at least one sample positive for 16S-PCR. However, sequences of the amplified 16S *rRNA* confirmed *Microthrix parvicella*, *Corynebacterium mastitidis* and *Corynebacterium macginleyi* in 3 animals (Table 4). 16S *rRNA* sequence of the lesion sample from a mouse, *Mastomys spp*, with Test ID S11, showed sequence similarity at 81% to the 16S *rRNA* of *M. ulcerans* Agy99 (accession Nº NR 074861, GenBank). Additionally, sequencing of the *IS2404* amplicon revealed 99% identity to *M. ulcerans* Agy99 partial, plasmid, pMUM001, GenBank accession Nº CP000325.1 (S3 Fig.), and was also positive for ER. We have not been able to determine the VNTR profile of this sample yet.

**Table 4:** Mycobacteria detection in organs of animals with lesions.

**Mycobacteria detection in organs of animals with lesions**							
**Test ID**	**Genus**	**community**	**Trapping site**	**site of lesion**	**16S-positive organs**	**ER-positive organs**	**Isolate; sequence match**
**B8**	*Rattus*	Bepotenten	Oda River	Right ear	St, K	none	K; *M. parvicella*
**W5**	*Mastomys*	Wromanso	house	Tail	SI, K, SP, LS, AS	none	NA
**W11**	*Mastomys*	Wromanso	house	Abdomen	SI, St, CS	none	SI; *Corynebacterium. mastitidis*
**S6**	*Mastomys*	Sukuumu	house	left thigh	St	none	St; *Corybacterium macginleyi* strain JCL-2
**S11**	*Mastomys*	Sukuumu	house	Tail	Lu, CS, LB	LB*	LB; *M. ulcerans* strain Agy99

Stomach(St), Kidney(K), Small intestine(SI), Spleen(SP), Lesion biopsy(LB), Lesion swab(LS), caecum(CS), lungs(Lu), anal swab(AS). NA, not amplified.

* Also positive for IS2404 (99% sequence identity to MU Agy99). 16S positivity was used to infer presence of *Mycobacterium* spp in organs but IS2404 sequencing was used to confirm presence of MU.

### Micro geo-distribution of genotypes suggests ponds harbour diverse *M. ulcerans* ecovars

**Figure 4:**
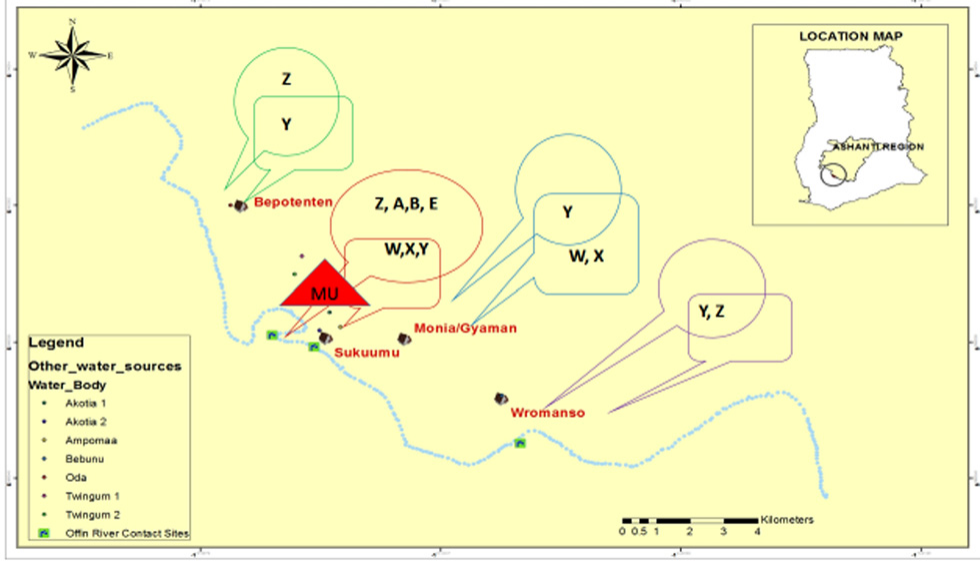
Community-based geographical distribution of MPM genotypes from humans and water bodies. The map of study communities was drawn using ArcMap 10. Rectangular and circular callouts contain genotypes of MU detected in humans and the environment respectively. Intersection of callouts contain genotypes common to both sources. Red triangle (in Sukuumu) represents MU-positive animal. Genotypes W, X, Y and Z were found both in the environment and human population. The Offin River is represented by blue dotted lines and contact sites on it are represented by green squares. Communities are represented by symbol for a house and other sampled water bodies are represented by colored dots as defined in the legend.

We overlapped human and environmental VNTR profiles within our study communities to observe MU genotype distribution (Fig. 4). Genotype Y intersects both environment and human populations in all communities. Genotype Z was detected only in environmental sources. A similar situation was observed in Monia where genotypes W and X were found in humans but not in environmental samples. Genotypes X and Y were detected in both the environment and in human infections in Wromanso. Sukuumu presented the most diverse genotypes, W, X and Y in both human and environmental samples and Z, A, B and E in the environment but not humans. The MU positive lesion sample, on the small mammal, was also detected in Sukuumu. In grouping genotypes from the three types of water bodies, we observed that ponds harboured 6/8 genotypes (X, Y, Z, A, B and E), rivers had 3/8 (Y, Z and OTS) and streams with 3/8 (W, Y and OTS). However, mycobacteria presence, particularly MU, was independent of the type of water body (P = 0.8081) when tested using the Chi-square contingency test for independence.

## Discussion

Environmental MPMs are of enormous medical importance due to their ability to cause opportunistic infections in humans and other vertebrates [16]. A better understanding of their ecology is crucial to controlling the spread of diseases they cause. Although few studies have typed environmental and clinical isolates of MU, the broader geographical distribution of these environmental pathogens against observed focal infections in human populations [5, 13] still poses a challenge to transmission studies. This is the first study that attempted to focally type MU samples from populations, the environment and small mammals in four endemic communities with the aim of tracking infections to MU-contaminated environments.

Water contact by individuals in our study communities were high although the presence of bore wells minimized the use of surface water for drinking purposes. Community members however used surface waters for bathing and washing purposes. With nine of the ten (9/10) water bodies sampled being positive for MU, frequent usage and exposure could be source of MU infections to the communities. All MU genotypes detected in patient samples collected from a community were also found in at least one community associated water body.

Mycobacteria presence was higher in biofilm (P = 0.0007). Similarly, MU presence was higher in biofilm, consistent with other reports [5, 6]. These suggest biofilm as the preferred microhabitat for MU within aquatic environments. AFB microscopy revealed clumps of MU bacilli in biofilm samples as compared to single bacilli in detritus. Thus, future attempts to culture MU from the environment could focus on concentrating biofilm. Additionally, we noticed that at least one sample from any type of water body tested positive for IS2404. Although a preliminary confirmation of MPMs, it suggests that MU and other MPMs may be found in most fresh water bodies, in endemic communities.

Using four loci, we increased the discrimination power of VNTR typing to obtain four MU genotypes with similar allelic profiles consistent with previous data [6, 14] and showed that VNTR typing for environmental samples could give varying allelic combinations. Currently, MU is the only reported MPM causing BU in humans except a few reported cases in some small mammals [16]. In this study, we first genotyped MU samples from humans and then compared the profiles to samples obtained from water bodies frequently used by inhabitants, including patients. This helped to properly match genotypes from the two sources and establish a possible source of infection within each community. At least one genotype was common to both sources, in all four endemic communities, suggesting their water bodies as likely sources of infection. Furthermore, these water bodies, particularly, rivers, may serve as vehicles for the dissemination of MU strains [32] and could partly explain why genotype Y was found in all communities. The four MU genotypes; W, X, Y and Z, from humans were identical to those obtained in the environment. Phylogenetic analysis clustered some environmental samples with human samples, and showed significant sequence homology to reported MU orthologs [9, 23, 24]. Additionally, genotypes A, B and E (*M. marinum* DL), similar to those reported by Williamson *et al*. [6], were found only in the environment. Ponds harboured most of the genotypes including *M. marinum* DL, genotype E. This data is parallel with studies associating the disease with stagnant or slow moving water bodies [6–8]. Repeats of 1 and 2, for MIRU1 and ST1 respectively, were constant for all human samples but varied for environmental samples. This is consistent with that reported by Hilty and colleagues [14] for MU strains in the Amansie West District, which is adjacent to the area of this study. Loci 6 and 19, each with repeats of 1 and 2, were the determinants of these genotypes, corroborating findings by similar studies [6, 21, 22, 33]. These data suggest that while it may be sufficient to use loci 6 and 19 to discriminate between MU strains from humans, a combination of all four is necessary to match human isolates to environmental ecovars in transmission studies. There have been no reported cases of co-infection and this was shown in a recent study [33]. However, Our VNTR typing, confirmed with sequencing, showed that one patient had two strains of MU, genotypes W and X.

Interestingly, in the current study, genotype Y, with a VNTR profile of (1, 2, 2, 1,) found in humans and water bodies, is identical to the purported *M. liflandii*, genotype, F (1, 2, 2, 1), reported by Williamson *et al*. [6]. *M. liflandii* has been reported as an environmental pathogen which causes infection in fishes and frogs and thus was considered a separate species [34]. However, recent sequencing and additional data on this pathogen suggest it is a *M. ulcerans* strain, *M. ulcerans* ecovar *liflandii* [35]. Thus, our data support this assertion and further suggest that genotype Y could be a pathogenic subtype of MU, which was previously considered *M. liflandii*, a frog, *Xenopus laevis*, pathogen [6]. However, this needs to be confirmed using cultured isolates.

Furthermore, genotype Y, was ubiquitous in soil, biofilm and detritus, in most water bodies. It was the only genotype common to the two sources, within each community. Genotype Z, identified in filtrands and biofilm, appeared to share similar ecology and epidemiology as genotype Y. This observation may suggest that mycobacteria with this genotype are able to survive and proliferate in different parts of the water body. Hence, patients could frequently be exposure to them, in relation to other strains. This may explain why they were detected in 50% of human samples, across the four communities. However, our data does not imply high transmissibility of Y/Z-type strains to humans nor their relative virulence as suggested by a recent study [33].

MU presence was observed to be independent of the type of water body (P = 0.8081) but we observed that ponds harboured most of the genotypes in all the four communities. Thus, while MU may be widely distributed in the environment [6], strains of the bacteria may have microhabitat preferences and this could account for focal transmission of the pathogen in some endemic communities [6, 13]. Our data, although preliminary, provides a good basis for studies to investigate ecological preferences of MU strains. Although genotypes A and B were present in the environment, they were not typed in human samples. Arguably, our sample number was low, however, it could be that strains with these genotypes occupy microhabitats where human contact were infrequent. Samples with similar genotypes have been shown to cause infection in humans [33]. Efforts to culture these environmental samples were unsuccessful, consistent with similar observations by Williamson *et al* [33], suggesting that existing laboratory culture conditions may be unfavourable for certain mycobacteria. We uncovered MPMs with VNTR profile OTS (1, 2, 7, ND) and ST1 repeats of 3 (*M. marinum*), which have not been previously reported, consolidating earlier statements of presence of pathogenic strains with propensity to cause infection in aquatic vertebrates and other small mammals [6, 9, 16, 35]. However, isolation and culturing of these strains are needed to substantiate these assertions.

Small mammals including possums and grasscutters have been observed to have lesions characteristic of BU [16, 20]. Field studies conducted in Benin to identify mammalian reservoirs detected other mycobacteria but not MU [19]. We report on the first detection of MU DNA from a tail lesion of a mouse, *Mastomys spp*, trapped in Sukuumu, a BU endemic community in Ghana. We observed a lesion, <1cm on the tail. ER-PCR of the lesion biopsy specimen was positive. Interestingly, sequencing of the 16S *rRNA* and *IS2404* amplicons showed greater than 81% and 98% identities respectively, to *M. ulcerans* strain Agy99. Our data, though preliminary, suggest that small mammals in BU endemic communities could be susceptible to MU infections. While these mammals may serve as reservoirs of MU, their definite role in transmission needs to be thoroughly investigated.

Several hypotheses for the transmission of MU bacilli from the environment to susceptible host have been put forth; bites from aquatic insects and mosquitoes, inoculation into open lesions and aerosolization of droplet nuclei [4, 36]. Activities including swimming, bathing and washing of clothes, irrigation, mining and certain agricultural activities may expose humans to MU. Individuals could be inoculated with MU, bacilli in biofilm, following a cut from a blade of grass. Within the aquatic system, there are complex interactions, where plants may provide substratum for MU to form biofilm [37], and aquatic vertebrates, e.g. fish may serve as reservoirs [38]. Additionally, a few aquatic insects have also been implicated in other proposed transmission routes [7]. Small mammals could infect humans with MU via bites, food and water contamination or handling during hunting. In turn, activities of these animals including drinking or searching for prey (fish, aquatic insects and food particles) around MU risk environments may expose them to infections [16]. Although the current study does not provide data on definite transmission route of MU or other MPMs, our results provide evidence to show that BU patients or individuals living in BU endemic communities could be most likely infected from MU-contaminated water bodies. Measures to control MU infections, Buruli ulcer, should therefore consider adopting a “OneHealth” approach, where various interdisciplinary efforts linking human, environmental and animal health sciences, can be used to decipher definite transmission routes of the pathogen.

### Conclusion

VNTR typing confirmed repeats previously reported by other studies and resolved the apparent homogeneity in MU isolates, in Ghana. In this study, we identified four *M. ulcerans* genotypes (W, X, Y, & Z) both in humans and sampled water bodies in the Amansie Central District of the Ashanti region, Ghana. Other previously reported *M. ulcerans* genotypes (A & B) and *M. marinum* DL, genotype E, were detected only in water bodies. Confirmation of repeat numbers by sequencing showed that certain MU strains harboured partial repeats and other MPMs had repeats not previously reported. Genetic comparisons and geo-distribution of genotypes were used to source track MU infections to specific water bodies. Our findings suggest that patients may have been infected from local water body sources, which also serve as reservoirs and vehicles for the dissemination of MU strains and other MPMs in endemic communities. Transmission of MU could involve several routes where humans have contact with risk environments. Further, we present evidence that small mammals within endemic communities could be susceptible to MU infections and may be acting as reservoirs. Thus, we support the hypothesis that transmission of MPMs, particularly, *M. ulcerans*, is dependent on the overlapping habitats of the pathogen and humans.

## Supporting Information

S1 Fig Comparison of AFB in two environmental samples. A) Shows a cord (clump of bacilli) of acid fast bacilli in a biofilm sample and B) shows individual bacilli (detritus sample) as shown by the arrow. (X1000 magnification)
(TIF)

S2 Fig Gel picture showing VNTR profiles of environmental and human *M. ulcerans* ecovars. FB1 is a human sample from Bepotenten and BAB-4 is a biofilm sample from Oda River, Bepotenten. Both typed genotype Y (1, 2, 2, 1). Additional band higher up in lane 4 of BAB-4 may suggest presence of other MPMs with more than 3 repeats for Locus 19. FS5 is a human sample taken from Sukuumu, which had two genotypes X (1, 1, 2, 2) and W (1, 1, 2, 1). Band sizes at lane, 340bp and 280bp, separated X and W respectively. Lanes 1, 2, 3 and 4 represent MIRU1, Locus 6, ST1 and Locus 19 respectively, for both upper and lower gels. Both gels were run with negative controls (Neg Ctrl).
(TIF)

S3 Fig Genetic relatedness of environmental samples using *IS2404* phylogeny. All environmental samples, together with the animal and human samples, formed a single cluster with reported *M. ulcerans* orthologs. The evolutionary history was inferred using the UPGMA method [39]. The optimal tree with the sum of branch length = 1.31639434. The percentage of replicate trees in which the associated taxa clustered together in the bootstrap test (1000 replicates) are shown next to the branches [40]. The reference sequences of the insertion sequence (*IS2404*) are represented with squares. Sequences of human and environmental samples are represented with triangles and circles respectively. The MU-positive animal sample (S11LB) is represented by the black dot.
(TIF)

S1 Table Activities around water bodies. (DOCX)

S2 Table List of primers used for PCR amplifications. (DOCX)

S3 Table Livelihood strategies. (DOCX)

S4 Table VNTR profiles and MPM strain designated genotypes of current study and from published data. W, X, Y and X are *M. ulcerans* designated genotypes from current study. A, B, C, and D are *M. ulcerans* designated genotypes from literature. Other published MPM genotypes; E is *M. marinum* DL, MPS is *M. pseudoshottsii*, MM is *M. marinum* and F is *M. liflandii*. ^a & c^ means identical, ^b^ means same genotype as in current study. ND, not done. Gh seq, Ghana sequence.
(DOCX)
