# Supplementary figures and images for: Source Tracking Mycobacterium ulcerans Infections in the Ashanti Region, Ghana
Source: PLoS Negl Trop Dis. 2015 Jan 22;9(1):e0003437. doi: 10.1371/journal.pntd.0003437 (PMC4303273; doi:10.1371/journal.pntd.0003437)

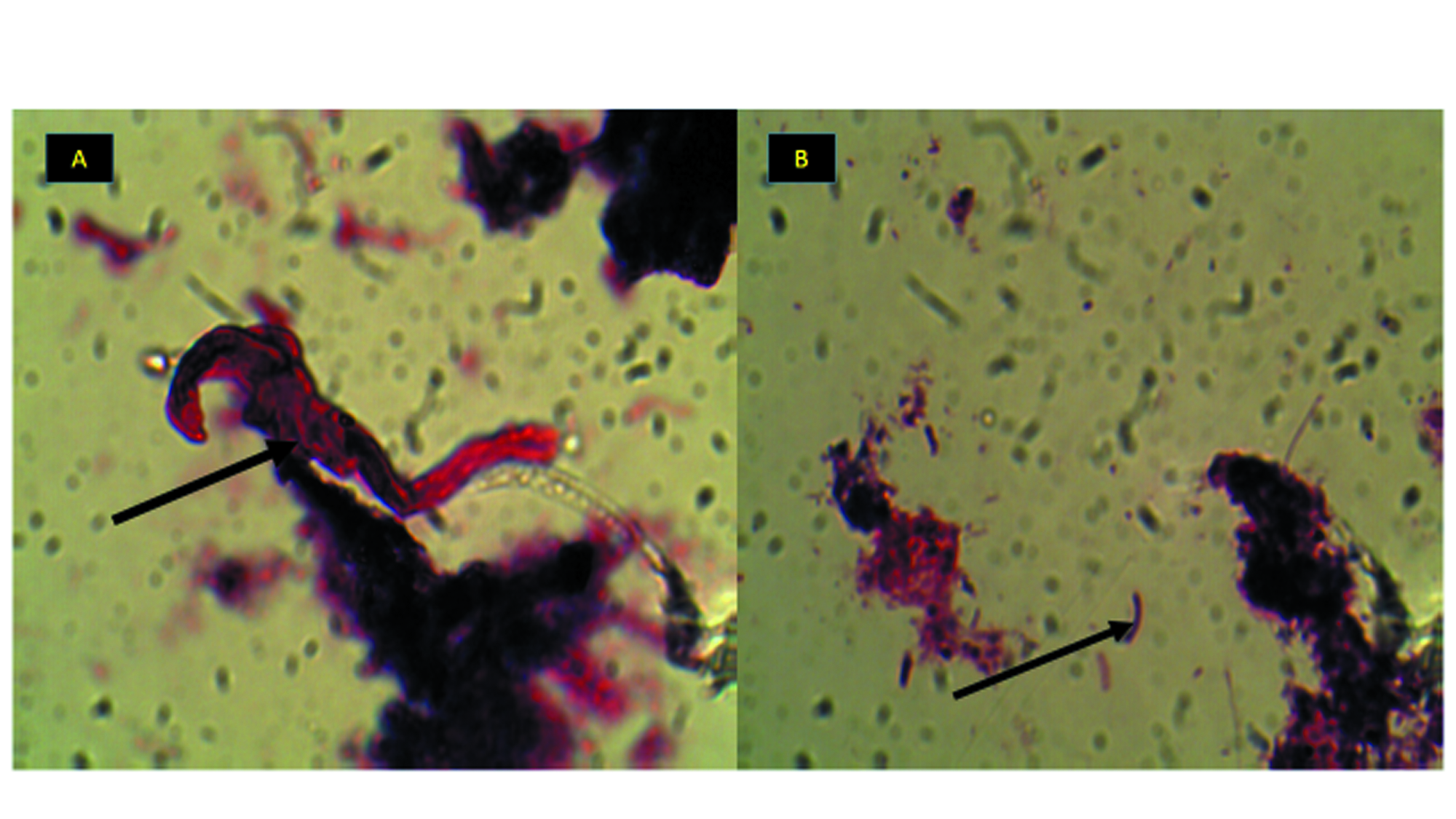

Supplement: S1 Fig — A) Shows a cord (clump of bacilli) of acid fast bacilli in a biofilm sample and B) shows individual bacilli (detritus sample) as shown by the arrow. (X1000 magnification) (TIF) [file pntd.0003437.s001.tif]
