# Supplementary material for: Source Tracking Mycobacterium ulcerans Infections in the Ashanti Region, Ghana
Source: PLoS Negl Trop Dis. 2015 Jan 22;9(1):e0003437. doi: 10.1371/journal.pntd.0003437 (PMC4303273; doi:10.1371/journal.pntd.0003437)
Supplement: S1 Table — (DOCX) [file pntd.0003437.s004.docx]

Table S1: Activities around water bodies

| Water Use (n=224) |  |  |
| --- | --- | --- |
|  | Frequency | Percentage |
| Bathing/Swimming | 100 | 44.64 |
| Drinking | 59 | 26.34 |
| Fishing | 125 | 55.80 |
| Laundry/Dishwashing | 42 | 18.75 |
| Others | 19 | 8.48 |
