# Supplementary material for: Source Tracking Mycobacterium ulcerans Infections in the Ashanti Region, Ghana
Source: PLoS Negl Trop Dis. 2015 Jan 22;9(1):e0003437. doi: 10.1371/journal.pntd.0003437 (PMC4303273; doi:10.1371/journal.pntd.0003437)
Supplement: S2 Table — (DOCX) [file pntd.0003437.s005.docx]

Table S2

| Primer | Forward and reverse sequences | Expected sizes (bp) | References |
| --- | --- | --- | --- |
| IS2404 (nest 1) | pGp1: 5’-AGGGCAGCGCGGTGATACGG-3’ | 400 | [[30](#_ENREF_30)] |
|  | pGp2: 5’-CAGTGGATTGGTGCCGATCGAG-3’ |  |  |
| IS2404 (nest 2) | pGp3: 5’-GGCGCAGATCAACTTCGCGGT-3’ | 200 | [[30](#_ENREF_30)] |
|  | pGp4: 5’-CTGCGTGGTGCTTTACGCGC-3 |  |  |
| 16S rRNA | PA: 5'AGAGTTTGATCCTGGCTCAG 3' | 600 | [[29](#_ENREF_29)] |
|  | MSHA: 5'AAAAAGCGACAAACCTACGAG 3' |  |  |
| ER | LM1 5’-CCTGACCGCCTACATCGCTTTG-3’ | 476 | Current study |
|  | LM2 5’-CAAACAGTGGGGACAGTTGGGC-3’ |  |  |
| Locus 6 | F-5’ GACCGTCATGTCGTTCGATCCTAGT 3’ | variable | [[6](#_ENREF_6)] |
|  | R-5’ GACATCGAAGAGGTGTGCCGTCT 3’ |  |  |
| Locus 19 | F-5’ CCGACGGATGAATCTGTAGGT 3’ | variable | [[6](#_ENREF_6)] |
|  | R-5’ TGGCGACGATCGAGTCTC 3’ |  |  |
| ST1 | F-5’ CTGAGGGGATTTCACGACCAG 3’ | variable | [[6](#_ENREF_6)] |
|  | R-5' CGCCACCCGCGGACACAGTCG 3' |  |  |
| MIRU1 | F-5’ GCTGGTTCATGCGTGGAAG 3’ | variable | [[6](#_ENREF_6)] |
|  | R-5’ GCCCTCGGGAATGTGGTT 3’ |  |  |
