# Supplementary material for: Source Tracking Mycobacterium ulcerans Infections in the Ashanti Region, Ghana
Source: PLoS Negl Trop Dis. 2015 Jan 22;9(1):e0003437. doi: 10.1371/journal.pntd.0003437 (PMC4303273; doi:10.1371/journal.pntd.0003437)
Supplement: S3 Table — (DOCX) [file pntd.0003437.s006.docx]

Table S3: Livelihood strategies

| Livelihood strategy (N=224) |  |  |
| --- | --- | --- |
|  | Frequency | Percentage |
| Agricultural farming | 215 | 95.98 |
| Animal Husbandry | 6 | 2.68 |
| Artisanship | 9 | 4.02 |
| Commerce | 40 | 17.86 |
| Fishing | 8 | 3.57 |
| Hunting | 7 | 3.13 |
| Others | 38 | 16.96 |
