# Supplementary material for: Source Tracking Mycobacterium ulcerans Infections in the Ashanti Region, Ghana
Source: PLoS Negl Trop Dis. 2015 Jan 22;9(1):e0003437. doi: 10.1371/journal.pntd.0003437 (PMC4303273; doi:10.1371/journal.pntd.0003437)
Supplement: S4 Table — W, X, Y and X are M. ulcerans designated genotypes from current study. A, B, C, and D are M. ulcerans designated genotypes from literature. Other published MPM genotypes; E is M. marinum DL, MPS is M. pseudoshottsii, MM is M. marinum and F is M. liflandii. a & c means identical, b means same genotype as in current study. ND, not done. Gh seq, Ghana sequence. (DOCX) [file pntd.0003437.s007.docx]

Table S4

| Designated genotype | VNTR Profiles | | | | Reference |
| --- | --- | --- | --- | --- | --- |
| Current study | MIRU1 | Locus 6 | ST1 | Locus 19 |  |
| W | 1 | 1 | 2 | 1 | Current study |
| X^a^ | 1 | 1 | 2 | 2 |  |
| Y^c^ | 1 | 2 | 2 | 1 |  |
| Z | 1 | 2 | 2 | 2 |  |
|  |  |  |  |  |  |
| Published data |  |  |  |  |  |
| A^b^ | 1 | 1 | 1 | 2 | [[6](#_ENREF_6), [33](#_ENREF_33)] |
| B^b^ | 3 | 1 | 1 | 2 |  |
| C | 3 | 1 | 2 | 2 |  |
| D^a^ | 1 | 1 | 2 | 2 |  |
| E^b^ | 1 | 2 | 1 | 2 |  |
| F^c^ | 1 | 2 | 2 | 1 |  |
| MPS/MM | 1 | 4 | 2 | 2 |  |
| Amansie west Strains |  |  |  |  |  |
| MU strain 1^b^ | 1 | ND | 2 | ND | [[14](#_ENREF_14)] |
| MU strain 2 | 3 | ND | 1 | ND |  |
| MU strain 3 | 3 | ND | 2 | ND |  |
| Gh seq MU strain^b^ | ND | 1 | ND | 2 | [[21](#_ENREF_21)] |
